# Positive Selection of a Serine Residue in Bat IRF3 Confers Enhanced Antiviral Protection

**DOI:** 10.1016/j.isci.2020.100958

**Published:** 2020-03-02

**Authors:** Arinjay Banerjee, Xi Zhang, Alyssa Yip, Katharina S. Schulz, Aaron T. Irving, Dawn Bowdish, Brian Golding, Lin-Fa Wang, Karen Mossman

**Affiliations:** 1 Michael DeGroote Institute for Infectious Disease Research, McMaster Immunology Research Centre, Department of Pathology and Molecular Medicine, McMaster University, Hamilton, ON L8S 4K1, Canada; 2 Department of Biology, McMaster University, Hamilton, ON L8S 4K1, Canada; 3 Programme in Emerging Infectious Disease, Duke-NUS Medical School, Singapore 169857, Singapore

**Keywords:** Biological Sciences, Immunology, Evolutionary Biology

## Abstract

Compared with other mammals, bats harbor more zoonotic viruses per species and do not demonstrate signs of disease on infection with these viruses. To counteract infections with viruses, bats have evolved enhanced mechanisms to limit virus replication and immunopathology. However, molecular and cellular drivers of antiviral responses in bats largely remain an enigma. In this study, we demonstrate that a serine residue in IRF3 is positively selected for in multiple bat species. IRF3 is a central regulator of innate antiviral responses in mammals. Replacing the serine residue in bat IRF3 with the human leucine residue decreased antiviral protection in bat cells, whereas the addition of this serine residue in human IRF3 significantly enhanced antiviral protection in human cells. Our study provides genetic and functional evidence for enhanced IRF3-mediated antiviral responses in bats and adds support to speculations that bats have positively selected for multiple adaptations in their antiviral immune responses.

## Highlights

• Serine 185 is positively selected for in multiple bat IRF3 sequences
• Serine 185 enhances IRF3-mediated antiviral responses in bat and human cells
• IRF3-S185 function is dependent on phosphorylation
• IFNAR complex is required for double-stranded RNA-mediated antiviral responses

## Graphical Abstract

**Figure:**
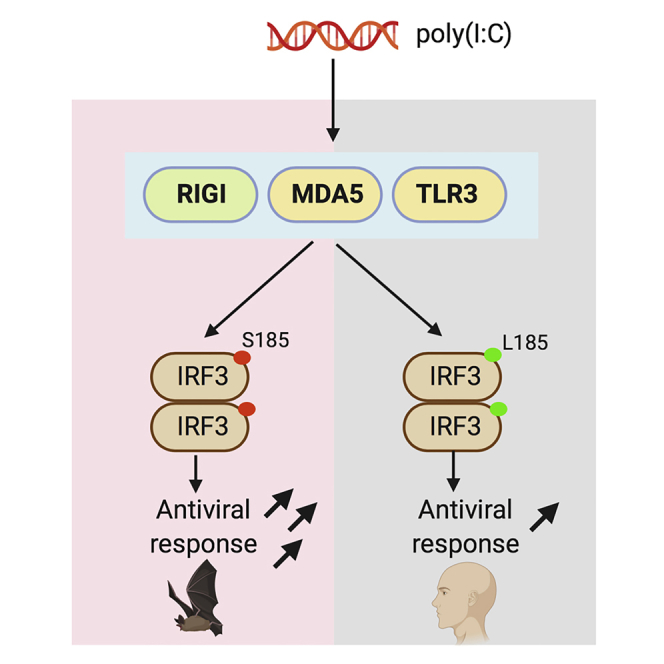

## Introduction

Bats are reservoirs of several emerging RNA viruses, such as filoviruses (ebolavirus and Marburg virus), paramyxoviruses (Nipah and Hendra viruses), and coronaviruses (severe acute respiratory syndrome [SARS] and Middle East respiratory syndrome [MERS] coronaviruses [CoVs]) that cause serious and often fatal disease in humans and agricultural animals (Anthony et al., 2017, Forbes et al., 2019, Ge et al., 2013, Swanepoel et al., 2007, Yang et al., 2019). More recently, SARS-CoV-2, which is causing the ongoing COVID-19 outbreak, was determined to be 96% similar at the genomic level to a bat CoV (Bat_CoV_RaTG13) that was detected in *Rhinolophus affinis* (Zhou et al., 2020). However, bats that are naturally or experimentally infected with these viruses do not demonstrate overt signs of disease (Munster et al., 2016, Hayman, 2016). These observations have led to studies that have explored innate and intrinsic antiviral immune responses in this intriguing mammalian order and the unique ability of bats to control virus infection-induced immunopathology (Pavlovich et al., 2018, Schountz et al., 2017).

In addition to identifying conserved features of the mammalian innate immune system in bats, recent studies have discovered novel adaptations in bat antiviral responses (Banerjee et al., 2020). These adaptations include constitutive expression of interferon alpha (IFNα) (Zhou et al., 2016), wider tissue distribution of interferon regulatory factor 7 (IRF7) (Zhou et al., 2014), stricter regulation of pro-inflammatory processes (Banerjee et al., 2017, Ahn et al., 2019), and atypical expression of interferon-stimulated genes (ISGs) (de La Cruz-Rivera et al., 2018, Hölzer et al., 2019). Most antiviral and innate immune signaling studies in bat cells have used surrogate virus (poly I:C, a synthetic double-stranded RNA molecule) and virus infections to stimulate downstream expression of IFNs and ISGs; however, the evolution and function of critical transcription factors, such as IRFs, and associated downstream antiviral signaling events remain an enigma.

IRF3 is a central transcription factor, and multiple antiviral signaling pathways converge on this molecule (Honda and Taniguchi, 2006, Honda et al., 2006). On sensing viral nucleic acids, pattern recognition receptors (PRRs) activate downstream signaling mediators, such as cellular kinases TANK binding kinase 1 (TBK1) and inhibitor of nuclear factor kappa-B kinase subunit epsilon (IKKϵ). Activated kinases phosphorylate serine residues in human IRF3 at positions 385, 386, 396, 398, and 402 to activate IRF3 (Panne et al., 2007). Activated IRF3 dimerizes and localizes to the nucleus of the cell to induce the expression of type I IFNs and downstream ISGs that induce an antiviral state in infected (autocrine) and neighboring (paracrine) cells (Kawai and Akira, 2006). A recent study in *Eptesicus fuscus* (big brown bat) cells demonstrated that IRF3 is essential for double-stranded (ds) RNA (polyI:C) and MERS-CoV infection-mediated stimulation of antiviral signaling pathways (Banerjee et al., 2019). However, residues important for IRF3 activation in bats have not been characterized. Considering the apparent asymptomatic co-existence of RNA viruses and bats (Maxmen, 2017, Wong et al., 2019), it is important to study the evolution of RNA virus detection and subsequent antiviral signaling in this mammalian order. Bats evolved/diverged from land mammals over 80 million years ago (Teeling et al., 2005, Simmons et al., 2008), and the prolonged arms race with some of these viruses, coupled with the unique ability to fly, have likely shaped their antiviral responses (O'shea et al., 2014, Zhang et al., 2013). Considering the important role of IRF3 in mediating downstream antiviral signaling events and the ability of multiple bat-borne RNA viruses to inhibit IRF3 activation in human cells (Lui et al., 2016, Ding et al., 2014, Chen et al., 2014), bats have likely evolved sophisticated mechanisms of IRF3 activation to mount a robust antiviral response to low levels of infection.

**Figure 1:**
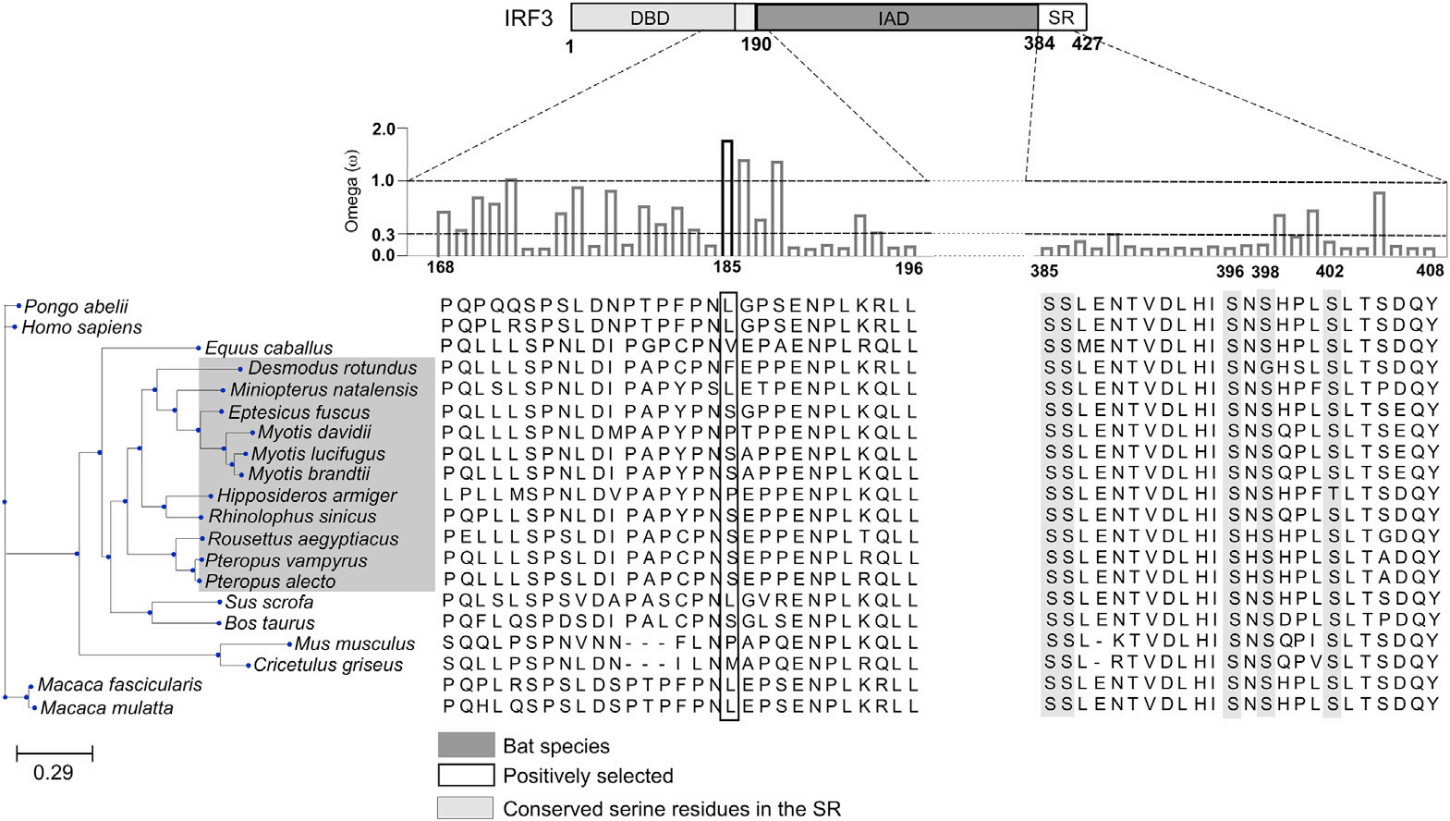
Positive Selection of Amino Acid Residue at the 185^th^ Position in bat IRF3 Functional domains of IRF3 are shown in the top panel above the alignment. The ratio of non-synonymous and synonymous amino acid substitutions is denoted by the omega value. Black bar indicates significant positive selection. Bat species are highlighted in dark gray. Conserved serine residues involved in human IRF3 activation are highlighted in light gray. The 185^th^ amino acid residue in the multiple sequence alignment is highlighted by the box. DBD, DNA-binding domain; IAD, IRF association domain; SR, serine-rich region. See also Figure S1 for details on computational analysis and Table S2 for accession numbers of IRF3 sequences.

In light of these discoveries and speculations, we hypothesized that co-existence with RNA viruses has imposed strong selective pressures on bat antiviral signaling molecules, resulting in robust antiviral responses to virus infection or immune activation signals. Since IRF3 is a key transcription factor in several virus-sensing pathways, we conducted computational and functional analyses of bat IRF3 across both suborders of bats, *Yinpterochiroptera* and *Yangochiroptera*. Sequence alignment of representative mammalian IRF3 sequences showed that serine (S) residues in the C-terminal serine-rich region are highly conserved (Figure 1). On performing further *in silico* analysis of IRF3 amino acid sequences, we identified that the amino acid residue at the 185^th^ position was positively selected for in multiple bat IRF3 sequences (Figure 1). Since serine residues play a major role in IRF3 activation (Panne et al., 2007), we studied the functional importance of the serine residue at the 185^th^ position (S185) in 7 of 11 bat IRF3 sequences.

## Results

### Serine 185 Is Positively Selected for in Multiple Bat Species

We conducted computational and functional analyses of bat IRF3 across both suborders of bats, *Yinpterochiroptera* and *Yangochiroptera*. Sequence alignment of representative mammalian IRF3 sequences showed that serine (S) residues in the C-terminal serine-rich region are highly conserved (Figure 1). On performing further *in silico* analysis of IRF3 amino acid sequences, we identified that the amino acid residue at the 185^th^ position was positively selected for in multiple bat IRF3 sequences (Figure 1). Since serine residues play a major role in IRF3 activation (Panne et al., 2007), we next studied the functional importance of the serine residue at the 185^th^ position (S185) in 7 of 11 bat IRF3 sequences.

### IRF3-S185 Induces a Robust Antiviral Response in Bat Cells from Two Suborders

**Figure 2:**
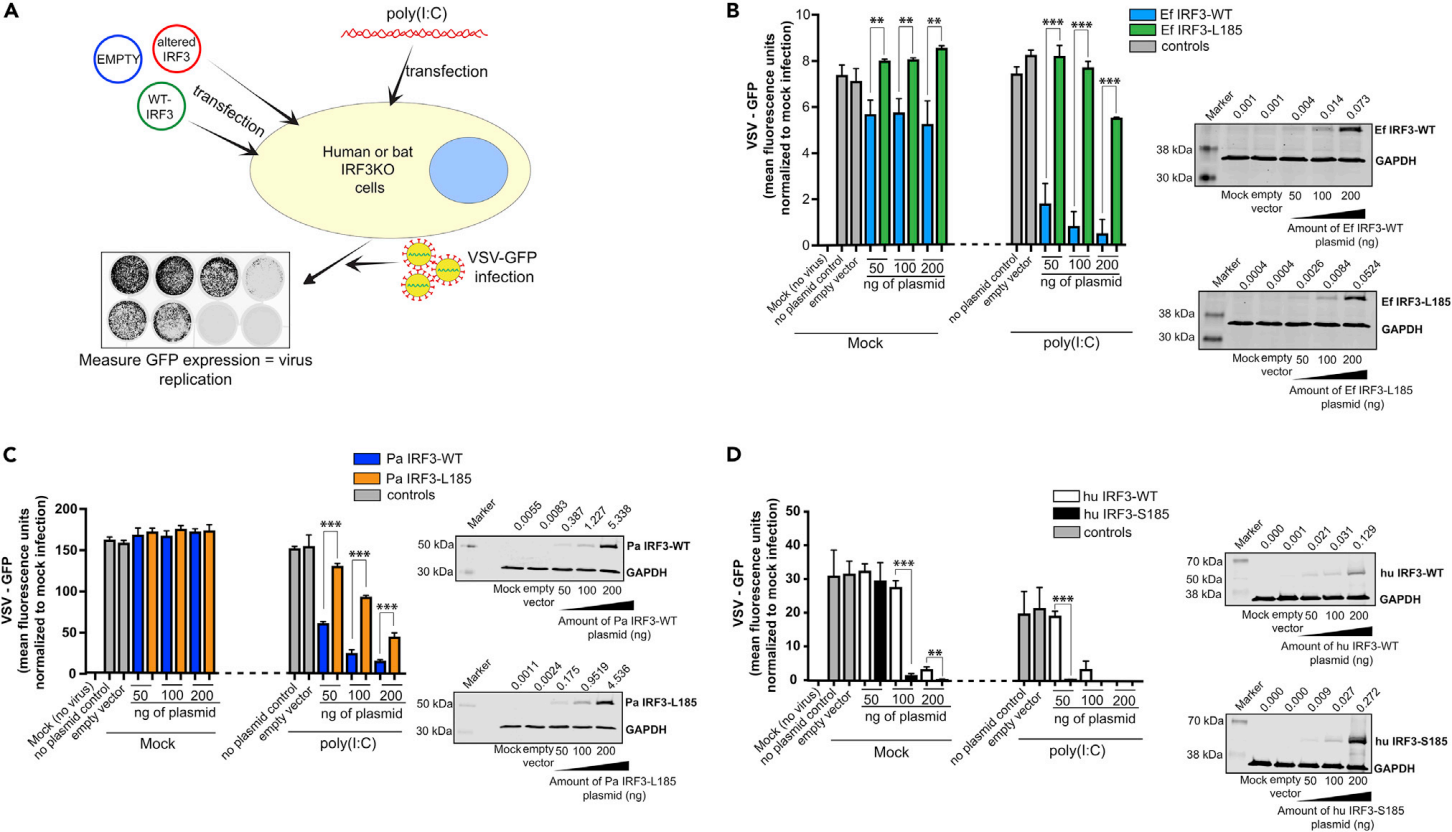
Human and Bat Cells Expressing IRF3-S185 Display Enhanced Antiviral Protection (A) Schematic representation of the experimental strategy. *IRF3* knockout (KO) bat and human cells were transfected with varying concentrations of wild-type (WT) or altered IRF3 expression plasmids for 24 h. The cells were then stimulated with poly(I:C) for 6 h, followed by infection with vesicular stomatitis virus (VSV) that was engineered to express green fluorescent protein (GFP). Nineteen hours after infection, GFP expression was measured as a surrogate for virus replication. (B) VSV-GFP replication in *E. fuscus IRF3* KO kidney cells (cr3-8) transfected with varying concentrations of plasmids expressing WT (S185) or altered (L185) *E. fuscus* IRF3 and mock treated or treated with poly(I:C) (n = 3). No plasmid and 200 ng of empty vector were used as transfection controls. Immunoblots: IRF3 protein levels in cr3-8 cells mock transfected, transfected with 200 ng empty vector (pcDNA), or transfected with varying concentrations of WT (S185) or altered (L185) IRF3 expression plasmids. (C) VSV-GFP replication in *P. alecto IRF3* KO kidney cells (PakiT03-4G) transfected with varying concentrations of plasmids expressing WT (S185) or altered (L185) *P. alecto* IRF3 and mock treated or treated with poly(I:C) (n = 3). No plasmid and 200 ng of empty vector were used as transfection controls. Immunoblots: IRF3 protein levels in PakiT03-4G cells mock transfected, transfected with 200 ng empty vector (pcDNA) or transfected with varying concentrations of WT (S185) or altered (L185) IRF3 expression plasmids. (D) VSV-GFP replication in human *IRF3* KO cells (THF-IRF3-KO) transfected with varying concentrations of plasmids expressing WT (L185) or altered (S185) human IRF3 and mock treated or treated with poly(I:C) (n = 3). No plasmid and 200 ng of empty vector were used as transfection controls. Immunoblots: IRF3 protein levels in THF IRF3 KO cells mock transfected, transfected with 200 ng empty vector (pcDNA) or transfected with varying concentrations of WT (L185) or altered (S185) IRF3 expression plasmids. Data are represented as mean ± SD, n = 3, ∗∗p < 0.01, ∗∗∗p < 0.001 (Student's t test). GFP expression is represented after normalization with mock infected cells. IRF3 protein expression and quantification data are expressed as a ratio of IRF3/GAPDH levels on top of the blots. Blots were quantified using Image Studio (LI-COR) (n = 3). KO, knockout; WT, wild-type; Ef, *E. fuscus*; Pa, *P. alecto*; Hu, human; NS, not significant.

To determine whether IRF3 is more competent in inducing robust antiviral protection due to the presence of S185, we compared the differences in antiviral response to surrogate virus infection [poly(I:C) stimulation] in bat and human cells expressing bat (S185) or human (L185) forms of IRF3, respectively. We generated *E. fuscus* and *P. alecto* wild-type (Ef IRF3-WT and Pa IRF3-WT) and altered (Ef IRF3-L185 and Pa IRF3-L185) IRF3 expression plasmids. We also generated wild-type (hu IRF3-WT) and altered (hu IRF3-S185) human IRF3 expression plasmids to determine if introducing S185 would enhance antiviral protection in human cells. To quantify the antiviral response in cells expressing wild-type or altered forms of IRF3, we performed bioassays using vesicular stomatitis virus (VSV) that was genetically engineered to express green fluorescent protein (VSV-GFP). VSV is known to infect cells from multiple species of mammals (Johannsdottir et al., 2009) and is very sensitive to IFN signaling, making it ideal for antiviral studies in cells from diverse mammalian species. In this study, we used *IRF3* deleted human fibroblast cells (THF-IRF3 KO cells) (Sali et al., 2015) and *IRF3* deleted kidney cells from two distantly related bat species, *E. fuscus* (*Yangochiroptera;* cr3-8 cells) (Banerjee et al., 2019) and *Pteropus alecto* (*Yinpterochiroptera;* PakiT03-4G cells). The use of IRF3-null cells allowed us to ectopically express wild-type and altered forms of IRF3 in a dose-dependent manner (Figure 2A).

To determine whether Ef IRF3-WT (S185) and Ef IRF3-L185 differed in their potential to activate antiviral signaling in *E. fuscus IRF3* deleted cr3-8 cells, we introduced increasing amounts of IRF3 expression plasmids in these cells (Figure 2B). We compared the extent of virus replication in Ef IRF3-WT and Ef IRF3-L185 expressing cr3-8 cells by quantifying the amount of GFP expressed by replicating VSV-GFP. Cr3-8 cells that expressed Ef IRF3-L185 displayed reduced antiviral protection compared with Ef IRF3-WT, both in the absence and presence of poly(I:C) stimulation (Figure 2B). Thus, replacing S185 with L185 in *E. fuscus* IRF3 significantly reduced poly(I:C)-induced antiviral protection in cr3-8 cells and led to higher levels of virus replication.

To determine if S185 in IRF3 was equally important for antiviral responses in a distantly related fruit bat, *P. alecto* (Figure 1), we expressed *P. alecto* WT (Pa IRF3-WT; S185) and altered (Pa IRF3-L185) IRF3 in *IRF3* deleted PakiT03-4G cells. Similar to what we observed in cr3-8 cells, expressing Pa IRF3-L185 in PakiT03-4G cells significantly reduced antiviral protection in these cells, relative to cells that expressed Pa IRF3-WT (Figure 2C). Thus, the presence of S185 in *E. fuscus* and *P. alecto* IRF3 is critical for a robust antiviral response in cells from these bats.

### Introducing S185 in Human IRF3 Enhances Antiviral Responses in Human Cells

To determine whether introducing a similar serine residue in human IRF3 could enhance antiviral responses in human cells, we introduced a complementary mutation in human IRF3 by replacing L185 with S185. We expressed wild-type (hu IRF3-WT; L185) and altered (hu IRF3-S185) human IRF3 in *IRF3* deleted human (THF-IRF3 KO) cells (Figure 2D). THF-IRF3 KO cells expressing hu IRF3-S185 were better protected against VSV-GFP in the absence or presence of poly(I:C) stimulation, compared with cells that expressed hu IRF3-WT (Figure 2D). Thus, introducing a serine residue at the 185^th^ position in human IRF3 significantly enhanced antiviral protection in human cells.

### IRF3-D185 Retains Enhanced Antiviral Signaling in Stimulated Bat and Human Cells

**Figure 3:**
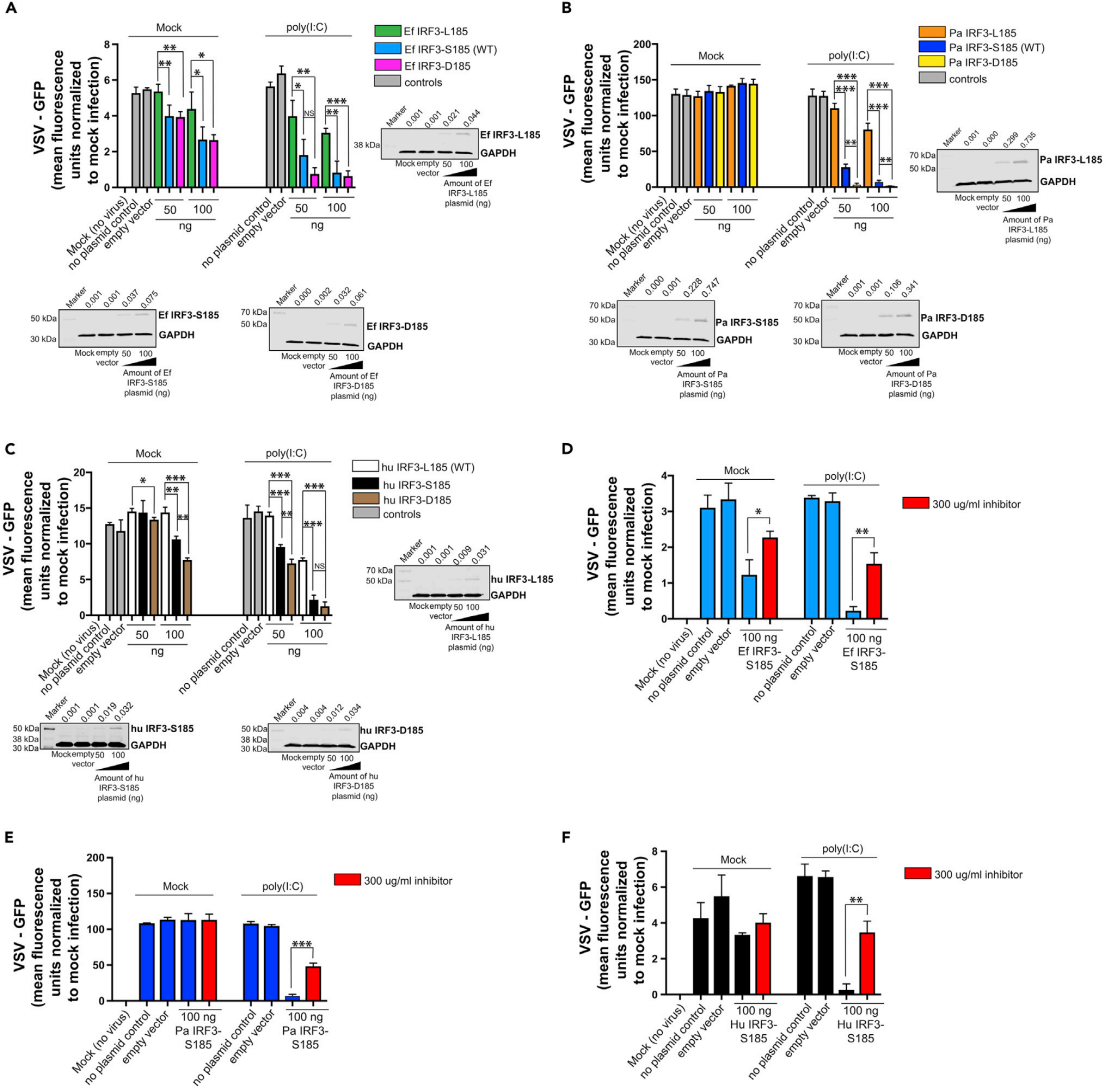
IRF3 S185- and D185-Expressing Bat and Human Cells Mount a Robust Antiviral Response to Double-Stranded RNA (A) VSV-GFP replication in *E. fuscus IRF3* KO kidney cells (cr3-8) transfected with varying concentrations of plasmids expressing L185, S185, or D185 forms of *E. fuscus* IRF3 and mock treated or treated with poly(I:C) (n = 3). No plasmid and 100 ng of empty vector were used as transfection controls. Immunoblots: IRF3 protein levels in cr3-8 cells mock transfected, transfected with 100 ng empty vector (pcDNA), or transfected with varying concentrations of L185, S185, and D185 IRF3 expression plasmids. (B) VSV-GFP replication in *P. alecto IRF3* KO kidney cells (PakiT03-4G) transfected with varying concentrations of plasmids expressing L185, S185, or D185 forms of *P. alecto* IRF3 and mock treated or treated with poly(I:C) (n = 3). No plasmid and 100 ng of empty vector were used as transfection controls. Immunoblots: IRF3 protein levels in PakiT03-4G cells mock transfected, transfected with 100 ng empty vector (pcDNA), or transfected with varying concentrations of L185, S185, or D185 IRF3 expression plasmids. (C) VSV-GFP replication in human *IRF3* KO cells (THF-IRF3-KO) transfected with varying concentrations of plasmids expressing L185, S185, or D185 forms of human IRF3 and mock treated or treated with poly(I:C) (n = 3). No plasmid and 100 ng of empty vector were used as transfection controls. Immunoblots: IRF3 protein levels in THF-IRF3-KO cells mock transfected, transfected with 100 ng empty vector (pcDNA), or transfected with varying concentrations of L185, S185, and D185 IRF3 expression plasmids. (D) VSV-GFP replication in *E. fuscus IRF3* KO kidney cells (cr3-8) transfected with 100 ng of plasmid expressing *E. fuscus* IRF3-S185 and mock treated or treated with 300 μg/mL of TBK1 and IKKϵ inhibitor. After treatment with the inhibitor, cells were mock stimulated or stimulated with poly(I:C) (n = 3). Normalized VSV-GFP levels in cells treated with TBK1 and IKKϵ inhibitor are denoted by red bars. No plasmid and 100 ng of empty vector were used as transfection controls. (E) VSV-GFP replication in *P. alecto IRF3* KO kidney cells (PakiT03-4G) transfected with 100 ng of plasmid expressing *P. alecto* IRF3-S185 and mock treated or treated with 300 μg/mL of TBK1 and IKKϵ inhibitor. After treatment with the inhibitor, cells were mock stimulated or stimulated with poly(I:C) (n = 3). Normalized VSV-GFP levels in cells treated with TBK1 and IKKϵ inhibitor are denoted by red bars. No plasmid and 100 ng of empty vector were used as transfection controls. (F) VSV-GFP replication in human *IRF3* KO cells (THF-IRF3-KO) transfected with 100 ng of plasmid expressing human IRF3-S185 and mock treated or treated with 300 μg/mL of TBK1 and IKKϵ inhibitor. After treatment with the inhibitor, cells were mock stimulated or stimulated with poly(I:C) (n = 3). Normalized VSV-GFP levels in cells treated with TBK1 and IKKϵ inhibitor are denoted by red bars. No plasmid and 100 ng of empty vector were used as transfection controls. Data are represented as mean ± SD, n = 3, ∗p < 0.05, ∗∗p < 0.01, ∗∗∗p < 0.001 (Student's t test). GFP expression is represented after normalization with mock infected cells. IRF3 protein expression and quantification data are expressed as a ratio of IRF3/GAPDH levels on top of the blots. Blots were quantified using Image Studio (LI-COR) (n = 3). KO, knockout; WT, wild-type; Ef, *E. fuscus*; Pa, *P. alecto*; Hu, human; NS, not significant. See also Figure S1.

Phosphorylation of serine residues in the C-terminal serine-rich region is known to regulate IRF3 activation in human cells (Panne et al., 2007). We next determined if the role of S185 in enhancing IRF3-mediated antiviral protection was dependent on phosphorylation. Since anti-phospho antibodies to S185 are not available, we substituted the serine residue at the 185^th^ position with aspartate (S185D mutation) in human and bat IRF3. Aspartate mimics the charge on a phosphorylated serine residue and has been used to study cellular functions that are modulated by phosphorylated serine residues in proteins (Leger et al., 1997). We repeated our bioassays and compared antiviral responses in cells expressing L185, S185 and D185 forms of IRF3. Indeed, human and bat IRF3-D185 conferred enhanced antiviral protection in human and bat cells, respectively, relative to IRF3-L185, suggesting that the activity of S185 is dependent on a charge that is similar to a phosphorylated serine residue (Figures 3A–3C). Interestingly, IRF3-D185 conferred enhanced protection in PakiT03-4G cells, relative to IRF3-S185-expressing cells that were stimulated with poly(I:C) (Figure 3B). Similarly, IRF3-D185 expressing THF-IRF3 KO cells were better protected from VSV-GFP, relative to IRF3-S185-expressing cells (Figure 3C; mock). Transfecting 50 ng of IRF3-D185-expressing plasmid also conferred better protection in THF-IRF3 KO cells than IRF3-S185 in the presence of poly(I:C) stimulation (Figure 3C). However, in the presence of poly(I:C), 100 ng of plasmid transfection of D185 and S185 forms of human IRF3 conferred comparable and significant protection in THF-IRF3 KO cells, relative to IRF3-L185 (Figure 3C). There were no significant differences in antiviral protection between S185 and D185 forms of IRF3 in cr3-8 cells (Figure 3A).

To further confirm if phosphorylation is critical for the activity of IRF3-S185, we used a human kinase inhibitor to block TBK1 and IKKϵ in cells expressing IRF3-S185 (Reilly et al., 2013, Yu et al., 2015). To determine if IRF3-S185-mediated antiviral responses in bat cells were dependent on TBK1 and IKKϵ-mediated phosphorylation, we first validated the cross-reactivity of the inhibitor in wild-type *E. fuscus* (Efk3B) and *P. alecto* (PakiT03) cells. We treated Efk3B and PakiT03 cells with varying concentrations of the inhibitor and stimulated the cells with poly(I:C) for 3 h. We used a cross-reactive phospho-IRF3 S396 antibody to detect phosphorylation of IRF3 (see Figure S1A). The inhibitor blocked phosphorylation of the 396^th^ serine residue, a marker of IRF3 activation, in cells from both species of bats in response to poly(I:C) stimulation (see Figures S1B and S1C). Next, we tested the effect of using the inhibitor in *IRF3* deleted bat cells expressing IRF3-S185. Ef and Pa IRF3-S185-expressing bat cells (cr3-8 and PakiT03-4G cells, respectively) that were treated with the inhibitor and stimulated with poly(I:C) had significantly higher levels of virus replication, relative to mock inhibitor-treated and poly(I:C)-induced IRF3-S185-expressing cells (Figures 3D and 3E). For cr3-8 cells expressing IRF3-S185, treating the cells with the inhibitor reduced basal levels of antiviral protection even in the absence of poly(I:C) (Figure 3D; mock). As observed in bat cells, inhibiting TBK1 and IKKϵ in THF-IRF3 KO cells expressing human IRF3-S185 significantly increased virus replication (Figure 3F).

### Wild-Type and IRF3-S185-Mediated Antiviral Responses in Bat and Human Cells Are Dependent on IFNAR Complex

**Figure 4:**
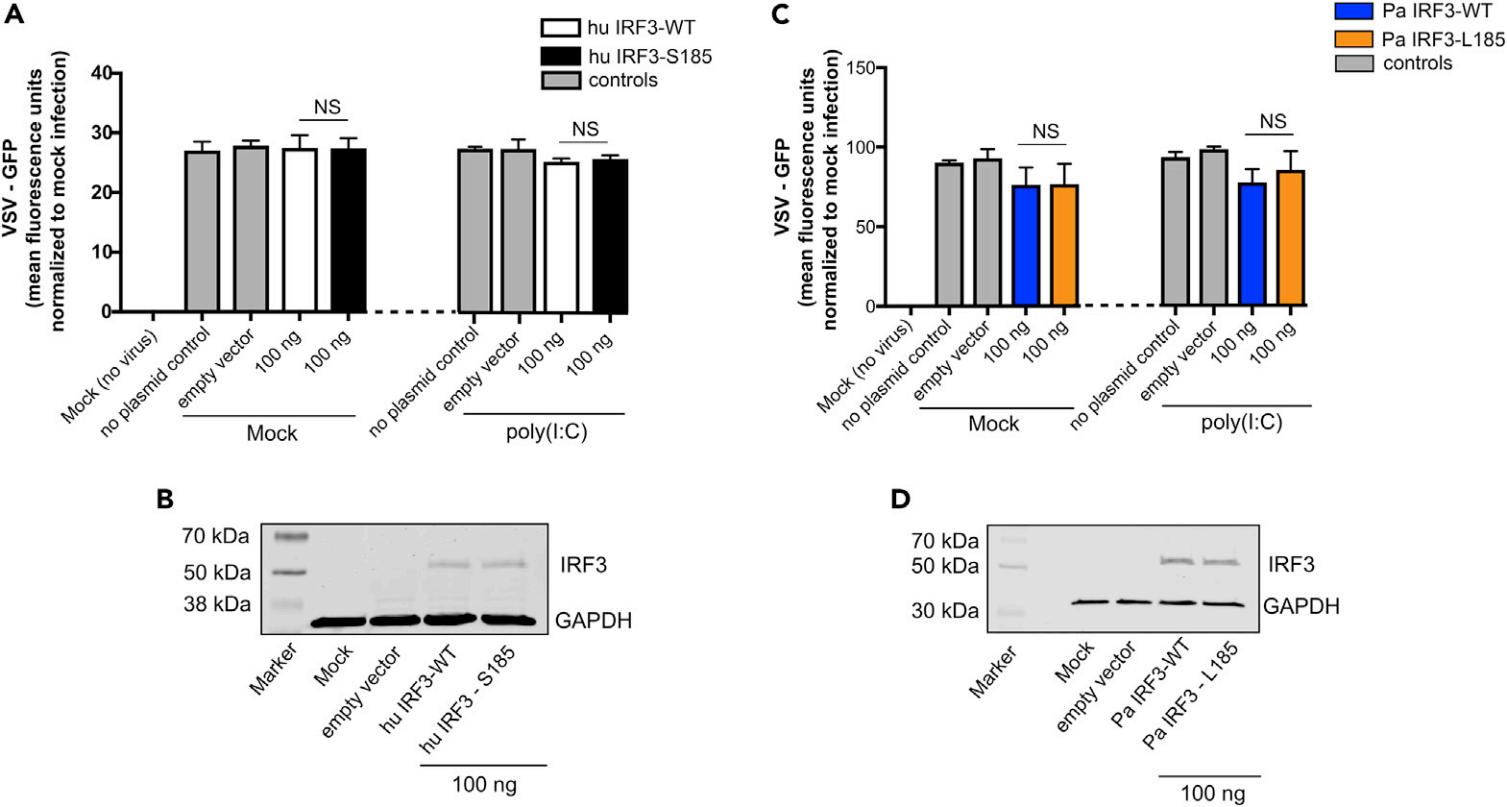
Wild-Type IRF3 and IRF3-S185-Mediated Antiviral Responses in Bat and Human Cells Are Dependent on the Expression of the IFNAR Complex (A) VSV-GFP replication in human *IRF3* and *IFNAR1* (THF-IRF3-IFNAR1 dKO) deleted cells transfected with 100 ng of plasmid expressing WT (L185) or altered (S185) human IRF3 and mock treated or treated with poly(I:C) (n = 3). No plasmid or 100 ng of empty plasmid (pcDNA) were used as transfection controls. (B) IRF3 expression in human *IRF3* and *IFNAR1* double knockout (THF-IRF3-IFNAR1 dKO) cells mock transfected, transfected with 100 ng empty vector (pcDNA), or transfected with 100 ng of WT (L185) or altered (S185) IRF3 expression plasmids. (C) VSV-GFP replication in *P. alecto IRF3* and *IFNAR2* (PakiT03-IFNAR2-IRF3-G6 dKO) deleted cells transfected with 100 ng of plasmid expressing WT (S185) or altered (L185) *P. alecto* IRF3 and mock treated or treated with poly(I:C) (n = 3). No plasmid or 100 ng of empty plasmid (pcDNA) were used as transfection controls. (D) IRF3 expression in *P. alecto IRF3* and *IFNAR2* double knockout (PakiT03-IFNAR2-IRF3-G6 dKO) cells mock transfected, transfected with 100 ng empty vector (pcDNA), or transfected with 100 ng of WT (S185) or altered (L185) IRF3 expression plasmids. Data are represented as mean ± SD, n = 3. GFP expression is represented after normalization with mock infected cells. KO, knockout; WT, wild-type; Ef, *E. fuscus*; Pa, *P. alecto*; Hu, human; NS, not significant.

Activation of IRF3 following an exogenous stimulus induces the expression of type I IFNs (Honda et al., 2006) and the subsequent expression of antiviral ISGs via binding to the IFN α/β receptors 1 and 2 complex (IFNAR1 and IFNAR2) (de Weerd et al., 2007). We and others have also shown that IRF3-mediated signaling can induce ISG expression independent of IFN production (Ashley et al., 2019, Noyce et al., 2011). To determine if antiviral protection observed in cells expressing S185 or L185 forms of IRF3 was dependent on type I IFN signaling, we repeated our bioassays (Figure 2A) in *IRF3* and *IFNAR1* double knockout (dKO) human cells (THF-IRF3-IFNAR1 dKO) and *IRF3* and *IFNAR2* dKO *P. alecto* (PakiT03-IFNAR2-IRF3-G6) cells. Expressing hu IRF3-WT (L185) or hu IRF3-S185 in THF dKO cells (Figure 4B) did not induce antiviral protection upon poly(I:C) stimulation (Figure 4A). Similarly, expressing Pa IRF3-WT (S185) or Pa IRF3-L185 in PakiT03-IFNAR2-IRF3-G6 dKO cells (Figure 4D) did not induce antiviral protection in response to poly(I:C) (Figure 4C). These data demonstrate that IRF3-L185 and S185-mediated antiviral responses to double-stranded RNA in human and bat cells are dependent on canonical IFN signaling via the IFNAR complex. The lack of antiviral protection in *IRF3* and *IFNAR* deleted human and bat cells demonstrate that antiviral protection in our bioassays are mediated through type I IFNs (Uzé et al., 1990).

## Discussion

Bats harbor many zoonotic RNA viruses and do not demonstrate signs of disease when they are naturally or experimentally infected with these viruses (Munster et al., 2016, Schuh et al., 2017, Amman et al., 2015). Multiple studies have demonstrated the ability of bat cells to produce antiviral IFNs and downstream ISGs; however, the role of key transcription factors, such as IRF3 in the antiviral signaling cascade has not been studied. In this study, we provide genetic and functional evidence that multiple bat IRF3 sequences have positively selected for a serine residue that confers enhanced antiviral protection in both bat and human cells. Interestingly, we also observed that *Desmodus rotundus* IRF3 sequence contained a phenylalanine residue at the 185^th^ position and a glycine residue at the 398^th^ position (Figure 1) and *Hipposideros armiger* IRF3 sequence contained a proline residue at the 185^th^ position and a threonine residue at the 402^nd^ position (Figure 1). As high-quality sequences and cell lines from these bats become available, it will be interesting to test the functional relevance of these mutations in bat IRF3 at the 185^th^ position and the serine-rich region.

We observed a decrease in the antiviral response in unstimulated *E. fuscus* (cr3-8) cells expressing IRF3-L185 (Figure 2B). These data suggest that S185 in bat IRF3 may contribute to higher basal levels of IFNs and associated antiviral protection in bat cells, as reported by Zhou et al. (2016). However, we did not observe an obvious similar response in unstimulated *P. alecto* cells (Figure 2C), highlighting the species diversity of bats (Teeling et al., 2005) and differences in cell types cultured from bats. In addition, we also observed that transfecting increasing concentrations of the IRF3 expression plasmid, in the absence of poly(I:C) stimulation (mock treated), did not induce strong antiviral protection in bat cells, unlike in unstimulated THF-IRF3 KO cells that were protected by higher concentrations of transfected plasmid alone (Figures 2B–2D). This observation is consistent with the recent finding that bats have evolved dampened DNA sensing and stimulator of IFN genes (STING)-mediated signaling to limit innate and intrinsic responses to self-DNA (Xie et al., 2018).

We observed that D185 form of IRF3 induced enhanced antiviral protection in response to poly(I:C) in PakiT03-4G cells, relative to S185 and L185 forms of IRF3 (Figure 3B). We observed a similar enhanced antiviral response in THF-IRF3 KO cells that were transfected with 50 ng of D185 [poly(I:C) stimulated] and 100 ng of D185 (mock stimulated), relative to cells transfected with similar concentrations of L185 and S185 IRF3 expression plasmids (Figure 3C). These data indicate that under certain circumstances, IRF3-D185 provides added antiviral protection from VSV, relative to L185 and S185 forms of IRF3. We speculate that D185 may aid in the phosphorylation of additional serine residues in the serine-rich region of IRF3 to enhance downstream antiviral responses. However, we did not observe a significant difference between S185 and D185 forms of IRF3 in cr3-8 cells (Figure 3A). Thus, although the data suggest that IRF3-D185 enhances antiviral response in human and *P. alecto* cells, relative to IRF3-S185, differences in IRF3 activation mechanisms may exist between different bat species.

Our data show that S185 enhances IRF3-mediated antiviral responses in human and bat cells and that this phenomenon is dependent on kinase-mediated activation of IRF3 in response to poly(I:C) treatment (Figures 3D and 3E). We also show for the first time that blocking bat TBK1 and IKKϵ using an inhibitor reduces phosphorylation of the 396^th^ serine residue in bat IRF3 (see Figures S1B and S1C) and subsequently dampens IRF3-S185-mediated antiviral protection against replicating VSV in bat cells (Figures 3D and 3E). It has been demonstrated that phosphorylation of S396 in human IRF3 alleviates autoinhibition and facilitates the phosphorylation of S385 and S386, thus amplifying the antiviral response (Panne et al., 2007). Similarly, phosphorylation of IRF3-S185 likely enhances phosphorylation of other serine residues in the serine-rich region that amplifies IRF3-mediated antiviral responses in bat and human cells. We also noted that treating bat cells with TBK1 and IKKϵ inhibitor did not restore VSV-GFP replication to levels observed in control cells (Figures 3D and 3E). It is possible that the kinase inhibitor is not as efficient in bat cells. Alternatively, we cannot rule out the presence of other kinases in bat cells that are capable of phosphorylating IRF3 in the absence of TBK1 and IKKϵ. Similarly, we observed that treating human cells with the kinase inhibitor did not restore virus replication to levels observed in control cells (Figure 3F). Since THF IRF3-KO cells were transfected with IRF3-S185 expressing plasmid prior to treatment with the inhibitor, the partial protection is likely due to plasmid-mediated upregulation of antiviral responses, which was observed in mock treated cells as well (Figure 3F). Further studies are required to identify the role of S185 in enhancing phosphorylation of additional serine residues in the serine-rich region of IRF3, along with any conformational changes that may be induced by the phosphorylation of S185 to facilitate additional phosphorylation events.

Loss of IRF3 has been linked to age-related cell senescence (Zhang et al., 2019), and a robust type I IFN response is associated with tumor regression and control (Hobeika et al., 1997). Bats display an exceptionally long lifespan (Foley et al., 2018, Huang et al., 2019, Wilkinson and Adams, 2019) and have evolved mechanisms that may mitigate tumor formation (Brook and Dobson, 2015). The role of IRF3 in aging and mitigation of tumorigenesis in bats is still speculative, but our data clearly demonstrate that IRF3 with S185 is a more potent inducer of antiviral responses in both bat and human cells. Future studies will elucidate on the possibility of leveraging knowledge from studies in bats to develop therapeutic strategies or enhanced therapeutic molecules for alternate mammalian species, such as humans.

## Limitations of the Study

Owing to the lack of anti-phospho antibodies to S185 in IRF3, we were unable to verify the phosphorylation of S185. The lack of cell lines and reagents from additional bat species did not allow us to explore the role of S185 and other mutations in the serine-rich region of IRF3 in other species of bats. As IRF3 deleted cell lines from other bat species become available, it will be interesting to identify species-specific adaptations and the role of S185 in antiviral responses against emerging bat-borne RNA viruses, such as filoviruses, paramyxoviruses, and coronaviruses, including the recently emerged SARS-CoV-2. Another limitation of this study is the use of one non-bat cell line from humans. As more IRF3 knockout cell lines from additional mammalian species are generated, it will be interesting to observe the effect of S185 on IRF3-mediated antiviral responses.

## Methods

All methods can be found in the accompanying Transparent Methods supplemental file.

## Supplemental Information

Document S1. Transparent Methods, Figure S1, and Tables S1 and S2
