# Supplementary material for: Positive Selection of a Serine Residue in Bat IRF3 Confers Enhanced Antiviral Protection
Source: iScience. 2020 Mar 2;23(3):100958. doi: 10.1016/j.isci.2020.100958 (PMC7075978; doi:10.1016/j.isci.2020.100958)
Supplement: Document S1. Transparent Methods, Figure S1, and Tables S1 and S2 [file mmc1.pdf]

iScience, Volume *23*

Supplemental Information

Positive Selection of a Serine Residue in Bat IRF3 Confers Enhanced Antiviral Protection

Arinjay Banerjee, Xi Zhang, Alyssa Yip, Katharina S. Schulz, Aaron T. Irving, Dawn Bowdish, Brian Golding, Lin-Fa Wang, and Karen Mossman

SUPPLEMENTAL ITEMS

FIGURE

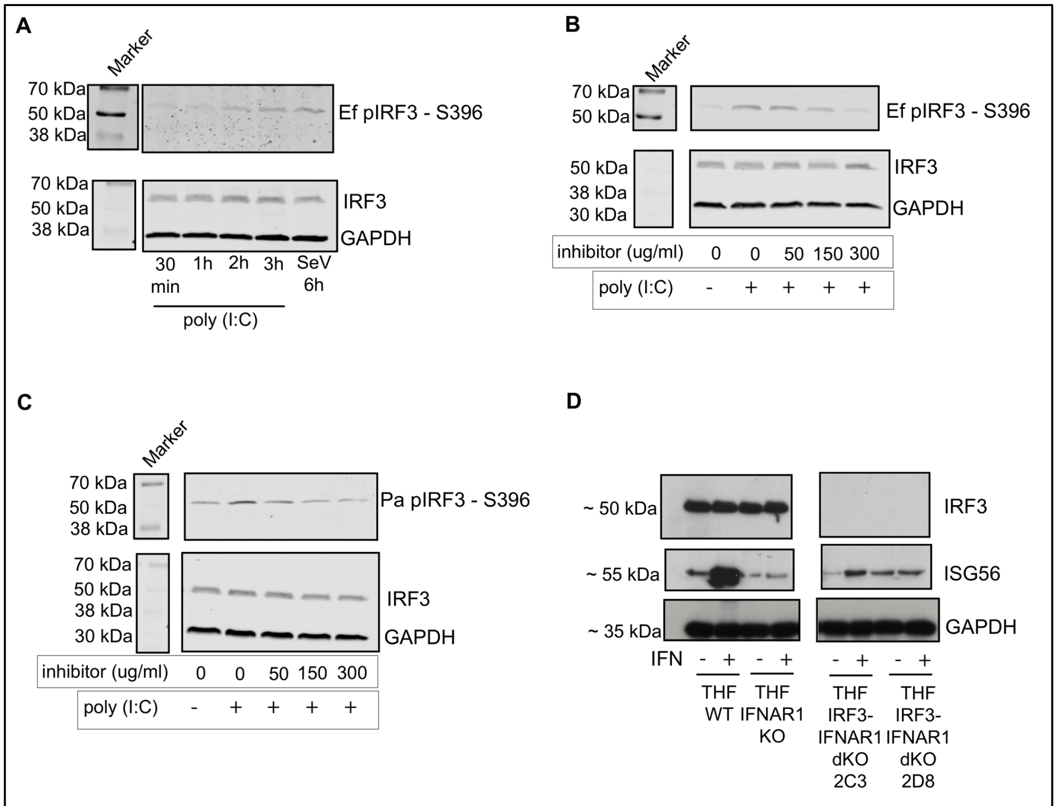

E

Model	np	lnL
M1	39	-8690.032497
M2	41	-8687.247593
M7	39	-8667.782341
M8	41	-8660.451760

Amino acid residue	Pr(w>1)	post mean +- SE for w
38 R	0.658	1.284 +- 0.360
161 G	0.553	1.193 +- 0.405
162 V	0.573	1.213 +- 0.391
175 N	0.796	1.388 +- 0.314
207 S	0.633	1.263 +- 0.369
234 S	0.957*	1.498 +- 0.204
235 G	0.606	1.242 +- 0.377
237 S	0.591	1.224 +- 0.397
316 C	0.659	1.286 +- 0.352
329 W	0.661	1.286 +- 0.352
376 T	0.563	1.201 +- 0.403
386 R	0.797	1.388 +- 0.321
400 S	0.559	1.198 +- 0.400
430 Q	0.912	1.469 +- 0.243

Figure S1. Phosphorylation of IRF3, efficacy of TBK1 and IKKε inhibitor in bat cells, details on computational analysis and validation of IRF3 KO THF cells, Related to Figures 1 and 3, and STAR METHODS. (A) Determination of IRF3-S396 phosphorylation using a cross-reactive human pIRF3-S396 antibody. Efk3B cells were treated with 100 ng of poly(I:C) and cell lysates were harvested at multiple time points. Efk3B cells were also infected with Sendai virus for 6 h and cell lysates were harvested for immunoblots. Immunoblots for the 396th phospho-serine residue in IRF3 (pIRF3-S396), IRF3 and GAPDH are shown. Lanes 2-5 were removed from this blot. The deletion between the ladder and lanes 6-10 is indicated by a space.

(B) Efk3B cells were mock treated or treated with increasing concentrations of TBK1 and IKKε inhibitor (Amlexanox), and mock stimulated or stimulated with 100 ng of poly(I:C). Cell lysates were harvested for immunoblots. Immunoblots for pIRF3-S396, IRF3 and GAPDH are shown. Lanes 2-4 were removed from this blot. The deletion between the ladder and lanes 5-9 is indicated by a space.

(C) PakiT03 cells were mock treated or treated with increasing concentrations of TBK1 and IKKε inhibitor (Amlexanox), and mock stimulated or stimulated with 100 ng of poly(I:C). Cell lysates were harvested for immunoblots. Immunoblots for pIRF3-S396, IRF3 and GAPDH are shown. Lanes 2-4 were removed from this blot. The deletion between the ladder and lanes 5-9 is indicated by a space.

(D) Related to STAR Methods. Validation of *IRF3* and *IFNAR1* knockout in THF-IRF3-IFNAR1 double knockout (dKO) cells. IRF3, ISG56 and GAPDH protein expression were detected by immunoblots in mock-treated cells or cells treated with human leukocyte interferon for 1 hour. THF WT and THF-IFNAR1 KO cells were used as positive and negative controls, respectively for IFN-induced ISG56 expression. Different portions of the gels are represented here, separated by spaces. The blots were developed using chemiluminescent technology (see methods). Approximate sizes of the proteins are indicated.

(E) Related to Figure 1. Using the Python ETE3 package, the aligned sequences were tested for positive selection using PAML models. The results for IRF3 are shown. Model M1 was tested against M2. These models differ, with the later having an extra parameter with omega (w) greater than zero (indicating positive selection). The significantly increased likelihood indicates that a model with positive selection fits better (left panel). Model M7 was tested against M8. These models have categories that follow a beta distribution, while M8 allows for positive selection. The significantly increased likelihood indicates positive selection fits better. Since the likelihood ratios were significant (left panel), posterior probabilities were used to identify sites under positive selection. The differences, M7/M8, via Bayes Empirical Bayes (BEB) analysis (Yang et al., 2005) identified positively selected sites that are shown in the right panel. The sites are numbered according to *Bos taurus* IRF3 sequence. The Serine (S) residue at position 234 (234 S) corresponds to site 185 in Figure 1 and has an omega value significantly >1.

Ef, *Eptesicus fuscus*; Pa, *Pteropus alecto*; pIRF3, phospho-IRF3; WT, wildtype; IFNAR, interferon α/β receptor; dKO, double knockout; IRF3, interferon regulatory factor 3, GAPDH, Glyceraldehyde 3-phosphate dehydrogenase; ISG56, Interferon-stimulated gene 56.

TABLES

Table S1. Primer and guide RNA sequences used in this study, Related to STAR Methods.

Cloning and site-directed mutagenesis	Forward primer	Reverse primer
Clone human IRF3	GCCGCTAGCGCCACCATGGGAACCCCAAAG	GCCCTCGAGTCAGGTCTCCCCAGG
Clone *E. fuscus* IRF3	GCCGCTAGCGCCACCATGGGATCCCAG	GCCGAATTCCTAGAAATCCATG
Clone *P. alecto* IRF3	GCCGCTAGCGCCACCATGGCTACCCCAAAGC	GCCCTCGAGCTAGAAATCCATGTCC
Site-directed mutagenesis - human IRF3-S185	ACTCCCTTCCCAAACAGTGGGCCCTCTGAGAAC	GTTCTCAGAGGGCCCACTGTTTGGGAAGGGAGT
Site-directed mutagenesis - *E. fuscus* IRF3-L185	GCTCCCTACCCAAACCTAGGACCCCCTGAAAAC	GTTTTCAGGGGGTCCTAGGTTTGGGTAGGGAGC
Site-directed mutagenesis - *P. alecto* IRF3-L185	GCTCCCTGCCCAAACCTAGAACCCCCTGAAAAC	GTTTTCAGGGGGTTCTAGGTTTGGGCAGGGAGC
Site-directed mutagenesis - human IRF3-D185	ACTCCCTTCCCAAACGACGGGCCCTCTGAGAAC	GTTCTCAGAGGGCCCGTCGTTTGGGAAGGGAGT
Site-directed mutagenesis - *E.*	GCTCCCTACCCAAACGACGGACCCCCTGAAAAC	GTTTTCAGGGGGTCCGTCGTTTGGGTAGGGAGC

fuscus IRF3-D185		
Site-directed mutagenesis - *P. alecto* IRF3-D185	GCTCCCTGCCCAAACGACGAACCCCCTGAAAAC	GTTTTCAGGGGGTTCGTCGTTTGGGCAGGGAGC
**Generating CRISPR KO cells**	**Guide RNA sequence**	
THF IRF3-IFNAR1 dKO cells	IFNAR1 gRNA: AAACACTTCTTCATGGTATG	
PakiT03 - 4G (IRF3 KO cells)	Pa-IRF3-gRNA1-F1: CACCGTTGGAAGCACGGCTTGCGGC	Pa-IRF3-gRNA1-R1: AAACGCCGCAAGCCGTGCTTCCAAC
PakiT03-IFNAR2-IRF3-G6 dKO cells	Pa-IRF3-gRNA1-F1: CACCGTTGGAAGCACGGCTTGCGGC	Pa-IRF3-gRNA1-R1: AAACGCCGCAAGCCGTGCTTCCAAC
**Validating CRISPR edited KO cells**	**Primers to verify deletion/mutation in guide RNA binding site**	
	**Forward primer**	**Reverse primer**
PakiT03 - 4G (IRF3 KO cells)	CACCGTTGGAAGCACGGCTTGCGGC	CACCGTTGGAAGCACGGCTTGCGGC
PakiT03-IFNAR2-IRF3-G6 (IFNAR2 and IRF3 dKO cells)	CACCGTTGGAAGCACGGCTTGCGGC	CACCGTTGGAAGCACGGCTTGCGGC
THF IRF3-IFNAR1 dKO	AACAGGAGCGATGAGTCTGTC	TGCGAAATGGTGTAAATGAGTCA

**Sequencing CRISPR edited *P. alecto* and THF cells**	**Guide RNA binding site sequence in CRISPR edited clonal cell population**	
PakiT03-4G (IRF3 KO)	IRF3-4G: -23 bp deletion in IRF3 coding sequences, homozygous CGCAGGTTGGACCATGGCTACCCCAAA GCCGAGGATCCTGCCCTGGCTAGTGTC GCAGCTGGACAGTGGGCAGCTGGAGGG CGTGGCATGGCTGAACGAGAGCCGCAC GCGCTTTCGCATCCCTTGGAAGCACGGC TTGCGGCAGGATGCCCAGCAGGAGGAC TTCGGCATCTTCCAGGTGCGCAGGAGC CAAGACTGGGCAAACACGGGGCGGGGC GGACTCCGAGGGCACTG	
PakiT03-IFNAR2-IRF3-G6 (IFNAR2 IRF3 dKO)	IRF3-/- IFNAR-/-: +1bp insertion in IRF3 coding sequence, homozygous CAGCTGGAGGGCGTGGCATGGCTGAAC GAGAGCCGCACGCGCTTTCGCATCCCTT GGAAGCACGGCTTGCCGGCAGGATGCC CAGCAGGAGGACTTCGGCATCTTCCAG GTGCGCAGGAG	
THF IRF3-IFNAR1 dKO	+2 bp insertion in IFNAR1 coding sequence TGACTCATTTACACCATTTCGCAAAGCT CAGATTGTGTCCTCCAGCAGTGAATGTT TAAATTTAAACGAATCGGAAGATCGGT AATTATTTGAT	

Table S2. NCBI accession numbers of IRF3 amino acid sequences used in phylogenetic and evolutionary analyses, Related to Figure 1.

**Mammal**	**IRF3 sequence accession number**
Pongo abelii	XP_009231174.2
Homo sapiens	XP_016882256.1
Equus caballus	XP_014585307.1
Desmodus rotundus	XP_024422099.1
Miniopterus natalensis	XP_016061535.1
Eptesicus fuscus	XP_008152570.1
Myotis davidii	XP_015420914.1
Myotis lucifugus	XP_014305320.1
Myotis brandtii	XP_005879595.1
Hipposideros armiger	XP_019513839.1
Rhinolophus sinicus	XP_019597087.1
Rousettus aegyptiacus	XP_015977865.1
Pteropus vampyrus	XP_011372830.1
Pteropus alecto	XP_006905084.1
Sus scrofa	NP_998935.1
Bos taurus	XP_024833997.1
Mus musculus	NP_058545.1
Cricetulus griseus	XP_027276506.1
Macaca fascicularis	XP_005589990.1
Macaca mulatta	NP_001129269.1

TRANSPARENT METHODS

Key Resources Table

REAGENT or RESOURCE	SOURCE	IDENTIFIER
**Antibodies**		
Rabbit anti-IRF3	Abcam	Cat#ab68481; RRID: AB_11155653
Rabbit anti-pIRF3-S396	Cell Signaling technology	Cat# 4947S; RRID: AB_823547
Mouse anti-GAPDH	EMD Milipore	Ca#AB2302; RRID: AB_10615768
Donkey anti-Rabbit 800	LI-COR	Cat#926-32213; RRID: 621848
Goat anti-mouse 680	LI-COR	Cat#925-68070; RRID: AB_2651128
Rabbit anti-IRF3 (FL-425)	Santa Cruz	Cat#sc-9082
Rabbit anti-ISG56	Laboratory of Dr. Ganes Sen	(Guo et al., 2000)
Mouse anti-GAPDH	Santa Cruz	Cat#sc-47724; RRID: AB_627678
Goat anti-mouse (peroxidase conjugated)	SIGMA	Cat#A8786; RRID: AB_258413
Goat anti-rabbit (peroxidase conjugated)	SIGMA	A0545; RRID: AB_257896
**Virus strain**		
Vesicular stomatitis virus - GFP (VSV-GFP)	Laboratory of Brian Lichty	(Leveille et al., 2011)
Sendai virus	Laboratory of Karen Mossman	(Noyce et al., 2006)
**Chemicals**		
Poly(I:C) (HMW)	InvivoGen	Cat#tlrl-pic
Lipofectamine 3000	Invitrogen	Cat#L3000015
Interferon from human leukocytes	Sigma-Aldrich	Cat#I4784
Amlexanox (TBK1 and IKKε inhibitor)	InvivoGen	Cat#inh-amx
**Critical Commercial Assays**		
QuikChange II site-directed mutageneisis	Agilent	Cat#200524-5

Q5 High-fidelity DNA polymerase	New England Biolabs	Cat#M0491S
**Experimental models: cell lines**		
cr3-8	Laboratory of Vikram Misra	(Banerjee et al., 2019)
THF-IRF3 KO	Laboratory of Victor DeFilippis	(Sali et al., 2015)
PakiT03 - 4G (IRF3 KO)	This study	NA
THF-IRF3-IFNAR1 dKO (double knockout)	This study	NA
PaKiT03-IFNAR2-IRF3-G6 dKO	This study	NA
Efk3B	Laboratory of Vikram Misra	(Banerjee et al., 2016)<br>RRID: CVCL_GZ34
PakiT03	Laboratory of Lin-fa Wang	(Zhang et al., 2017)<br>RRID: CVCL_DR89
PakiT03-IFNAR2-4A	Laboratory of Lin-fa Wang	(Zhang et al., 2017)
THF	Laboratory of Victor DeFilippis	(DeFilippis et al., 2010)
HEK293T	Laboratory of Brian D. Lichty	(Wang et al., 2016)
THF-IFNAR1 KO	Laboratory of Victor DeFilippis	(Pryke et al., 2017)
**Oligonucleotides**		
Primers for IRF3 cloning and mutagenesis, see Table S1	This study	NA
**Recombinant DNA**		
pcDNA3.1	ThermoFisher Scientific	Cat#V79020
pcDNA3.1 hu IRF3-WT	This study	NA

pcDNA3.1 hu IRF3-L185S	This study	NA
pcDNA3.1 Ef IRF3-WT	This study	NA
pcDNA3.1 Ef IRF3-S185L	This study	NA
pcDNA3.1 Pa IRF3-WT	This study	NA
pcDNA3.1 Pa IRF3-S185L	This study	NA
pcDNA3.1 hu IRF3-L185D	This study	NA
pcDNA3.1 Ef IRF3-S185D	This study	NA
pcDNA3.1 Pa IRF3-S185D	This study	NA
psPAX2	Addgene	Cat#12260
pMD2.G	Addgene	Cat#12259
plentiCRISPR hygro IFNAR	Laboratory of Victor DeFilippis	(Pryke et al., 2017)
**Softwares and algorithms**		
Prism software	GraphPad	https://www.graphpad.com
Image studio	LI-COR	https://www.licor.com/bio/image-studio/
Guide RNA design resources	Broad Institute	http://tools.genome-engineering.org

CONTACT FOR REAGENT AND RESOURCE SHARING

Dr. Karen Mossman (mossk@mcmaster.ca) and Dr. Lin-Fa Wang (linfa.wang@duke-nus.edu.sg).

COMPUTATIONAL ANALYSIS

The longest IRF3 isoform for each of eleven bat species was selected from the NCBI nucleotide database . A group of diverse species of mammals were selected to serve as outgroups. These included primates (*Homo sapiens*, *Macaca mulatta*, *Macaca fascicularis* and *Pongo abelli*), rodents (*Mus musculus*, and *Cricetulus griseus*), *Equus ferus, Bos taurus* and *Sus scrofa*. The alignments were carried out using MAFFT (version 7 with the G-INS-i (globalpair –maxiterate 1000) option) (Katoh et al., 2002). The alignment was manually checked using NCBI's multiple sequence alignment (MSA) viewer and checked to ensure that all codons were correctly aligned. RAxML HPC (version 8.2) was used to generate phylogenetic trees using amino acid sequences (Stamatakis, 2014). Phylogenetic trees were generated using the WAG matrix (GAMMA model). For each tree, 1000 bootstraps were generated. A majority consensus (>50%) tree was then calculated from the bootstrap trees. PAML CodeML v4.5 was automated by ETE3 package under Python 2.7 environment (Yang, 2007, Yang, 1997). Branch lengths were calculated by

CodeML. Site selection models (M1a, M2a, M7, M8) and branch-site model as specified by CodeML were tested. For the branch-site model, each branch, which consists of a single species was tested for positive selection. A likelihood ratio test was used to test the difference between the alternate and null hypothesis. M1 is the nearly neutral model, and was the null hypothesis that was tested against M2, which incorporates positive selection. Model M7 incorporates a beta distributed selection values while M8 adds a class of sites with positive selection. Model M8 was tested against the null hypothesis M7. Since the likelihood ratios were significant (see supplementary figure S1E; left panel), posterior probabilities were used to identify sites under positive selection. The differences, M7/M8, via Bayes Empirical Bayes (BEB) analysis (Yang et al., 2005) identified positively selected residues (see supplementary figure S1E; right panel).

EXPERIMENTAL MODEL

Viruses and Cell lines

THF, THF-IRF3 knockout (KO), THF-IFNAR1 KO, THF-IRF3-IFNAR1 double-KO (dKO) human fibroblast cells and HEK 293T cells were maintained in Dulbecco's modified Eagle medium (DMEM; Sigma) supplemented with L-Glutamine (Sigma), 10% fetal bovine serum (FBS; Sigma) and penicillin/streptomycin (ThermoFisher Scientific). Efk3B (wildtype cells) and cr3-8 (*E. fuscus* IRF3KO kidney) cells were maintained in DMEM supplemented with GlutaMax (Gibco), 10% FBS and penicillin/streptomycin. PakiT03, PakiT03-4G (IRF3 KO), PakiT03-IFNAR2-4A and PakiT03-IFNAR2-IRF3-G6 dKO cells (*P. alecto* kidney cells) were maintained in DMEM supplemented with GlutaMax (Gibco), 10% FBS and penicillin/streptomycin. All cells were incubated at 37°C in humidified, 5% CO_2 incubators. Cell lines were periodically tested for mycoplasma contamination. Stocks of genetically engineered vesicular stomatitis virus (VSV-GFP) carrying a GFP expression cassette (Noyce et al., 2011) were stored at -80°C. Virus stocks were thawed once and used for an experiment. A fresh vial was used for each experiment to avoid repeated freeze-thaws.

Generation and validation of CRISPR knockout cells

THF-IRF3-IFNAR1 double-KO (dKO) cells were generated by knocking out *IFNAR1* in previously published THF-IRF3 KO cells (DeFilippis et al., 2006). Lentivirus transduction was used to express Cas9 and guide RNA (gRNA) targeting *IFNAR1* in THF-IRF3 KO cells. Briefly, previously published transfer vector (plentiCRISPR hygro IFNAR) containing *IFNAR1* guide RNA (gRNA) and Cas9 (Pryke et al., 2017) (a gift from Victor DeFilippis' laboratory), and packaging plasmids psPAX2 (Addgene) and pMD2.G (Addgene) were transfected into HEK 293T cells using Lipofectamine 3000 (Invitrogen) (see Table S1 for gRNA sequence). 6 h post transfection, the transfection mixture was replaced with complete media containing 1% BSA. 60 h post transfection, the supernatant was harvested and virus was concentrated by ultracentrifugation. THF-IRF3 KO cells were infected with the lentivirus in complete media containing 0.1% polybrene. The following day, cells were passaged and seeded in 10 cm dishes in complete media containing hygromycin. 10 days later, single cell clones were selected and cultured over time. To characterize THF-IRF3-IFNAR1 dKO cells, we were unable to validate the knockout of IFNAR1 by immunoblots since commercially available antibodies did not cross-react with human IFNAR1. To overcome this limitation, we performed immunoblots to demonstrate the loss of IFNAR1-mediated downstream IFNα signalling and ISG56 expression in these cells (see supplementary figure S1D). Briefly, THF-WT, THF-IFNAR1 KO and THF-IRF3-IFNAR1 dKO cells were treated with interferon from human leukocytes (Sigma) for 1 h and cell lysates were harvested for immunoblots. 25 μg of total protein per sample was analyzed

by immunoblot for ISG56 and IRF3 protein expression. THF IRF3-IFNAR1 dKO clone 2D8 was used for subsequent studies.

For PakiT03-4G (IRF3KO) and PakiT03-IFNAR2-IRF3-G6 dKO cells, guide RNA design, vector construction, transfection, single cell screening and validation were done as previously described (Zhang et al., 2017). Parental *P. alecto* kidney cells (PakiT03) were used to generate *IRF3* knockout cells (PakiT03-4G). Similarly, previously published *P. alecto IFNAR2* knockout (PakiT03-IFNAR2-4A) cells (Zhang et al., 2017) were used to generate *IFNAR2* and *IRF3* double knockout cells (PakiT03-IFNAR2-IRF3-G6 dKO). To generate guide RNA targeting *IRF3*, exon sequence of *IRF3* was submitted to an online software (http://tools.genome-engineering.org) to obtain potential gRNA targets (see Table S1 for gRNA sequences). The top hits were further subjected to BLAST (NCBI) analysis against *P.alecto* genome to exclude off-target effects. Two gRNA sequences with high score and specificity were chosen for plasmid construction. The pSpCas9 (BB)-2A-GFP plasmid was used as a vector as previously published (Ran et al., 2013). *P. alecto* immortalized kidney (PaKiT03) cell lines (Crameri et al., 2009) were seeded in 6-well plates at a concentration of 8×10^5 cells/well and transfected with 1.5 µg of plasmid using Lipofectamine 3000 (Life Technologies, Carlsbad, CA, USA) as per manufacturer's recommendation. Two days after transfection, cells were sorted using FACSAria III (BD Biosciences). GFP-positive clones were collected and seeded in 96-well plates at a concentration of approximately 2 cells/well. One week later, single colonies of cells were selected for further validation using fluorescent capillary gel electrophoresis. Briefly, genomic DNA from clonal cell populations was extracted using QuickExtract solution (Epicentre) and genetic modifications were verified by PCR, followed by sequencing as previously described (Zhang et al., 2017) (see Table S1). In addition, THF-IRF3-KO and THF-IRF3-IFNAR1 dKO cells were validated using functional assays as part of this study (Figures 2D and 4A). Similarly, PakiT03-4G and PakiT03-IFNAR2-IRF3-G6 dKO cells were also validated by immunoblots and functional assays as part of this study (Figures 2C, 4C and 4D).

METHOD DETAILS

Plasmid construction

RNA was extracted from wildtype THF (human fibroblast), Efk3B (*E. fuscus* kidney cells) and PakiT03 (*P. alecto* kidney cells) cells using the RNeasy RNA extraction kit (Qiagen). cDNA was prepared from 500 ng of RNA using iScript gDNA clear cDNA synthesis kit (Bio-Rad). IRF3 coding sequences were amplified using a high-fidelity Q5 DNA polymerase (New England BioLabs) and species-specific primers with restriction sites (see Table S1 for primers). IRF3 amplicons were cloned into pcDNA3.1 (ThermoFisher Scientific) expression plasmid using restriction digestion (New England Biolabs), followed by ligation with T4 DNA ligase (Invitrogen). Restriction enzymes NheI and EcoRI (New England Biolabs) were used to digest *E. fuscus* IRF3 amplicon. NheI and XhoI (New England Biolabs) were used to digest human and *P. alecto* IRF3 amplicons. All plasmid constructs were confirmed by sequencing (Mobix).

Site-directed mutagenesis

Site-directed mutagenesis was performed using the QuikChange II site-directed mutagenesis kit (Agilent). Wang and Malcolm's modification of the manufacturer's protocol was used for this assay (Wang W, 1999). See Table S1 for primer information. Mutations were confirmed by sequencing (Mobix).

Transfection
IRF3 expression plasmids and poly(I:C) were transfected using Lipofectamine 3000 reagent as per manufacturer's recommendation (Invitrogen). Varying concentrations of IRF3 expression plasmids or 200 ng of empty (pcDNA 3.1) control plasmid were transfected for 24 hrs. After 24 hrs, media on the cells was replaced with fresh growth media and cells were either mock transfected or transfected with poly(I:C) for 6 hrs. 10 ng poly(I:C) was used for *E. fuscus* (cr3-8) and human (THF and all CRISPR/clonal derivatives) cell lines. After optimizing poly(I:C)-mediated antiviral responses in *P. alecto* cells (PakiT03 and all CRISPR/clonal derivatives), an optimal concentration of 100 ng was used for all studies in cells from this bat.

Virus infection and quantification
2 x 10^5 cells/well were seeded in 12-well plates and incubated overnight at 37°C with 5% CO_2. Following IRF3 or empty plasmid transfection for 24 hrs and subsequent 6 hrs of poly(I:C) or mock stimulation, cells were either mock infected or infected with VSV-GFP in serum-free media. Human and *E. fuscus* cells were infected with 5.5 x 10^4 $TCID_{50}$/ml of VSV-GFP. *P. alecto* cells were infected with 2.75 x 10^3 $TCID_{50}$/ml of VSV-GFP. For IRF3-S396 phosphorylation assay in *E. fuscus cells,* Efk3B cells were infected with 80 HA units of Sendai virus. Infected cells were incubated at 37°C for 1 hr with gentle rocking every 15 minutes. After 1 hr, virus inoculum was aspirated and Minimum Essential Medium (MEM) with Earle's salts (Sigma) containing 2% FBS and 1% carboxymethyl cellulose (CMC; Sigma) was added on the cells. The plates were incubated for 19 hrs (VSV-GFP) at 37°C and green fluorescent protein (GFP) levels were measured using a typhoon scanner (Amersham). Sendai virus infected cells were incubated for 6 hrs and cell lysates were processed for immunoblots.

Immunoblots
Cells were seeded at a concentration of 2 × 10^5 cells/well in 12-well plates and transfected with varying concentrations of IRF3 expression plasmids. Cells were harvested 24 hrs after transfection. Samples were denatured in a reducing sample buffer and analyzed on a reducing gel. Proteins were blotted from the gel onto polyvinylidene difluoride (PVDF) membranes (Immobilon, Milipore) and detected using primary and secondary antibodies. Primary antibodies used were: 1:1000 mouse anti-GAPDH (EMD Milipore; Catalogue number: AB2302; RRID: AB_10615768), 1:1000 rabbit anti-IRF3 (Abcam; Catalogue number: ab68481; RRID: AB_11155653) and 1:1000 rabbit anti-pIRF3-S396 (Cell Signaling technology; Catalogue number: 4947S; RRID: ab_823547). Secondary antibodies used were: 1:5000 donkey anti-rabbit 800 (LI-COR Biosciences; Catalogue number: 926-32213; RRID: 621848) and 1:5000 goat anti-mouse 680 (LI-COR Biosciences; Catalogue number: 925-68070; RRID: AB_2651128). Blots were observed and imaged using Image Studio (LI-COR Biosciences) on the Odyssey CLx imaging system (LI-COR Biosciences). For the detection of ISG56, GAPDH and IRF3 in THF and CRISPR/Cas9 modified THF cells (Figure S1D), enhanced luminol-based chemiluminescence was used as previously described (Noyce et al., 2006). Primary antibodies used were: 1:1000 rabbit anti-IRF3 (Santa Cruz; Catalogue number: sc-9082; RRID: AB_2264929), mouse anti-GAPDH (Santa Cruz; Catalogue number: sc-47724; RRID: AB_627678) and rabbit anti-ISG56 (gift from Dr. Ganes Sen). Peroxidase conjugated goat anti-rabbit (Sigma; Catalogue number: A0545; RRID: AB_257896) and goat anti-mouse (Sigma; Catalogue number: A8786; RRID: AB_258413) secondary antibodies were used for the chemiluminescent immunoblots. Films were scanned on a typhoon scanner (Amersham).

TBK1 and IKKε inhibitor treatment

1.5 x 10^5 cells/well were seeded in 12-well plates and incubated at 37ºC and 5% CO_2. 24 hrs after seeding cells, 100 ng of IRF3-S185 expression plasmids were transfected into cells. After 24 hrs, media was changed and cells were wither mock treated with DMSO or treated with various concentrations of the inhibitor (Amlexanox, InvivoGen) for 1 hr at 37ºC. After 1 hr, cells were mock treated or transfected with poly(I:C). 3-6 hrs after poly(I:C) transfection, cells were harvested for immunoblots or infected with VSV-GFP.

QUANTIFICATION AND STATISTICAL ANALYSIS

Immunoblot quantification

Immunoblot bands were quantified using Image Studio (LI-COR Biosciences).

Statistical Analysis

Data analysis were performed using SPSS statistics package version 21. All data are shown as Mean ± SD. Statistical analysis was performed using Student's t test with two-tailed, 95% confidence. P values less than 0.05 were considered statistically significant (* $p < 0.05$, ** $p < 0.01$ and *** $p < 0.001$). 'n' represents number of experimental replicates that were carried out and are specified in the figure legends.

REFERENCES

BANERJEE, A., FALZARANO, D., RAPIN, N., LEW, J. & MISRA, V. 2019. Interferon Regulatory Factor 3-Mediated Signaling Limits Middle-East Respiratory Syndrome (MERS) Coronavirus Propagation in Cells from an Insectivorous Bat. *Viruses,* 11.

BANERJEE, A., RAPIN, N., MILLER, M., GRIEBEL, P., ZHOU, Y., MUNSTER, V. & MISRA, V. 2016. Generation and Characterization of Eptesicus fuscus (Big brown bat) kidney cell lines immortalized using the Myotis polyomavirus large T-antigen. *J Virol Methods,* 237**,** 166-173.

CRAMERI, G., TODD, S., GRIMLEY, S., MCEACHERN, J. A., MARSH, G. A., SMITH, C., TACHEDJIAN, M., DE JONG, C., VIRTUE, E. R., YU, M., BULACH, D., LIU, J. P., MICHALSKI, W. P., MIDDLETON, D., FIELD, H. E. & WANG, L. F. 2009. Establishment, immortalisation and characterisation of pteropid bat cell lines. *PLoS One,* 4**,** e8266.

DEFILIPPIS, V. R., ROBINSON, B., KECK, T. M., HANSEN, S. G., NELSON, J. A. & FRUH, K. J. 2006. Interferon regulatory factor 3 is necessary for induction of antiviral genes during human cytomegalovirus infection. *J Virol,* 80**,** 1032-7.

DEFILIPPIS, V. R., SALI, T., ALVARADO, D., WHITE, L., BRESNAHAN, W. & FRUH, K. J. 2010. Activation of the interferon response by human cytomegalovirus occurs via cytoplasmic double-stranded DNA but not glycoprotein B. *J Virol,* 84**,** 8913-25.

GUO, J., PETERS, K. L. & SEN, G. C. 2000. Induction of the human protein P56 by interferon, double-stranded RNA, or virus infection. *Virology,* 267**,** 209-19.

KATOH, K., MISAWA, K., KUMA, K. & MIYATA, T. 2002. MAFFT: a novel method for rapid multiple sequence alignment based on fast Fourier transform. *Nucleic Acids Res,* 30**,** 3059-66.

LEVEILLE, S., GOULET, M. L., LICHTY, B. D. & HISCOTT, J. 2011. Vesicular stomatitis virus oncolytic treatment interferes with tumor-associated dendritic cell functions and abrogates tumor antigen presentation. *J Virol,* 85**,** 12160-9.

NOYCE, R. S., COLLINS, S. E. & MOSSMAN, K. L. 2006. Identification of a novel pathway essential for the immediate-early, interferon-independent antiviral response to enveloped virions. *J Virol,* 80**,** 226-35.

NOYCE, R. S., TAYLOR, K., CIECHONSKA, M., COLLINS, S. E., DUNCAN, R. & MOSSMAN, K. L. 2011. Membrane perturbation elicits an IRF3-dependent, interferon-independent antiviral response. *J Virol,* 85**,** 10926-31.

PRYKE, K. M., ABRAHAM, J., SALI, T. M., GALL, B. J., ARCHER, I., LIU, A., BAMBINA, S., BAIRD, J., GOUGH, M., CHAKHTOURA, M., HADDAD, E. K., KIRBY, I. T., NILSEN, A., STREBLOW, D. N., HIRSCH, A. J., SMITH, J. L. & DEFILIPPIS, V. R. 2017. A Novel Agonist of the TRIF Pathway Induces a Cellular State Refractory to Replication of Zika, Chikungunya, and Dengue Viruses. *MBio,* 8.

RAN, F. A., HSU, P. D., WRIGHT, J., AGARWALA, V., SCOTT, D. A. & ZHANG, F. 2013. Genome engineering using the CRISPR-Cas9 system. *Nat Protoc,* 8**,** 2281-2308.

SALI, T. M., PRYKE, K. M., ABRAHAM, J., LIU, A., ARCHER, I., BROECKEL, R., STAVEROSKY, J. A., SMITH, J. L., AL-SHAMMARI, A., AMSLER, L., SHERIDAN, K., NILSEN, A., STREBLOW, D. N. & DEFILIPPIS, V. R. 2015. Characterization of a Novel Human-Specific STING Agonist that Elicits Antiviral Activity Against Emerging Alphaviruses. *PLoS Pathog,* 11**,** e1005324.

STAMATAKIS, A. 2014. RAxML version 8: a tool for phylogenetic analysis and post-analysis of large phylogenies. *Bioinformatics,* 30**,** 1312-3.

WANG, F., ALAIN, T., SZRETTER, K. J., STEPHENSON, K., POL, J. G., ATHERTON, M. J., HOANG, H. D., FONSECA, B. D., ZAKARIA, C., CHEN, L., RANGWALA, Z., HESCH, A., CHAN, E. S. Y., TUINMAN, C., SUTHAR, M. S., JIANG, Z., ASHKAR, A. A., THOMAS, G., KOZMA, S. C., GALE, M., JR., FITZGERALD, K. A., DIAMOND, M. S., MOSSMAN, K., SONENBERG, N., WAN, Y. & LICHTY, B. D. 2016. S6K-STING interaction regulates cytosolic DNA-mediated activation of the transcription factor IRF3. *Nat Immunol,* 17**,** 514-522.

WANG W, M. B. 1999. Two-stage PCR protocol allowing introduction of multiple mutations, deletions and insertions using QuikChange Site-Directed Mutagenesis. *Biotechniques,* 26**,** 680-682.

YANG, Z. 1997. PAML: a program package for phylogenetic analysis by maximum likelihood. *Bioinformatics,* 13**,** 555-556.

YANG, Z. 2007. PAML 4: phylogenetic analysis by maximum likelihood. *Mol Biol Evol,* 24**,** 1586-91.

YANG, Z., WONG, W. S. & NIELSEN, R. 2005. Bayes empirical bayes inference of amino acid sites under positive selection. *Mol Biol Evol,* 22**,** 1107-18.

ZHANG, Q., ZENG, L. P., ZHOU, P., IRVING, A. T., LI, S., SHI, Z. L. & WANG, L. F. 2017. IFNAR2-dependent gene expression profile induced by IFN-alpha in Pteropus alecto bat cells and impact of IFNAR2 knockout on virus infection. *PLoS One,* 12**,** e0182866.
